# (*E*)-1-(2-Nitro­ethen­yl)naphthalene

**DOI:** 10.1107/S1600536809037623

**Published:** 2009-09-26

**Authors:** Lin-Hai Jing

**Affiliations:** a School of Chemistry and Chemical Engineering, China West Normal University, Nanchong 637002, People’s Republic of China

## Abstract

The title mol­ecule, C_12_H_9_NO_2_, adopts a *trans* configuration about the olefinic double bond. The dihedral angle between the naphthalene ring system (r.m.s. deviation = 0.012 Å) and the nitro­ethenyl group (r.m.s. deviation = 0.032 Å) is 12.66 (5)°. The mol­ecules are linked into a two-dimensional network parallel to the *bc* plane by C—H⋯O hydrogen bonds. The substituted benzene rings in adjacent networks are stacked with a centroid–centroid distance of 3.6337 (11) Å, indicating π–π inter­actions.

## Related literature

For general background to β-nitro­olefins, see: Barrett & Graboski (1986 ▶). For the synthesis, see: Cheng *et al.* (2007 ▶).
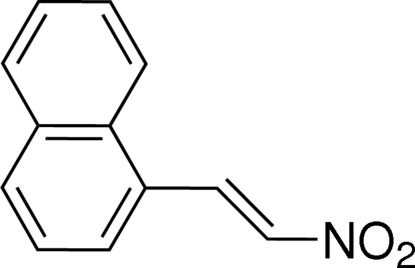


## Experimental

### 

#### Crystal data

C_12_H_9_NO_2_
*M* *_r_* = 199.20
Orthorhombic, 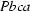

*a* = 7.2670 (14) Å
*b* = 13.741 (3) Å
*c* = 19.127 (4) Å
*V* = 1909.9 (6) Å^3^
*Z* = 8
Mo *K*α radiation
μ = 0.10 mm^−1^
*T* = 93 K
0.40 × 0.33 × 0.13 mm

#### Data collection

Rigaku SPIDER diffractometer
Absorption correction: none
14290 measured reflections
2179 independent reflections
2019 reflections with *I* > 2σ(*I*)
*R*_int_ = 0.030

#### Refinement

*R*[*F* ^2^ > 2σ(*F* ^2^)] = 0.048
*wR*(*F* ^2^) = 0.099
*S* = 1.00
2179 reflections
136 parameters
H-atom parameters constrained
Δρ_max_ = 0.30 e Å^−3^
Δρ_min_ = −0.20 e Å^−3^

### 

Data collection: *RAPID-AUTO* (Rigaku/MSC, 2004 ▶); cell refinement: *RAPID-AUTO*; data reduction: *RAPID-AUTO*; program(s) used to solve structure: *SHELXS97* (Sheldrick, 2008 ▶); program(s) used to refine structure: *SHELXL97* (Sheldrick, 2008 ▶); molecular graphics: *XP* in *SHELXTL* (Sheldrick, 2008 ▶); software used to prepare material for publication: *SHELXL97*.

## Supplementary Material

Crystal structure: contains datablocks global, I. DOI: 10.1107/S1600536809037623/ci2915sup1.cif

Structure factors: contains datablocks I. DOI: 10.1107/S1600536809037623/ci2915Isup2.hkl

Additional supplementary materials:  crystallographic information; 3D view; checkCIF report

## Figures and Tables

**Table 1:** Hydrogen-bond geometry (Å, °)

*D*—H⋯*A*	*D*—H	H⋯*A*	*D*⋯*A*	*D*—H⋯*A*
C2—H2⋯O2^i^	0.95	2.58	3.3919 (19)	144
C7—H7⋯O2^ii^	0.95	2.52	3.4261 (19)	159
C12—H12⋯O2^i^	0.95	2.57	3.464 (2)	158

Symmetry codes: (i) 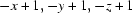; (ii) 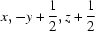.

**Fig. 1.**
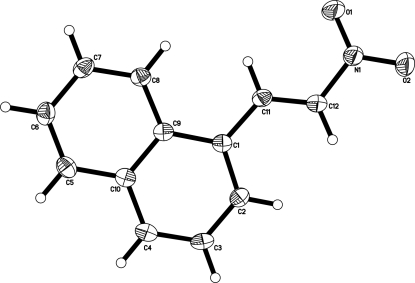
The molecular structure of the title compound, showing 50% probability displacement ellipsoids and the atomic numbering.

## supplementary crystallographic information

### Comment

β-Nitroolefins are a class of useful and versatile building blocks in organic synthesis (Barrett & Graboski, 1986). The author reports here, the crystal structure of the title compound.

Bond lengths and angles in the title molecule are normal. The molecule adopts a trans configuration about the olefinic double bond (Fig. 1). The naphthalene ring system is planar, with a maximum deviation of 0.021 (1) Å for atom C1. The dihedral angle between the C1-C9 and N1/O1/O2/C11/C12 planes is 12.66 (5)°. The molecules are linked into a two-dimensional network parallel to the *bc* plane by C—H···O hydrogen bonds (Table 1).

### Experimental

The title compound was synthesized according to the method reported in the literature (Cheng *et al.*, 2007). Yellow single crystals suitable for X-ray diffraction were obtained by slow evaporation of a methanol solution.

### Refinement

All H atoms were placed in calculated positions, with C-H = 0.95 Å, and refined using a riding model, with *U*_iso_(H) = 1.2*U*_eq_(C).

### Crystal data

**Table:**

C_12_H_9_NO_2_	*F*(000) = 832
*M**_r_* = 199.20	*D*_x_ = 1.386 Mg m^−^^3^
Orthorhombic, *P**b**c**a*	Mo *K*α radiation, λ = 0.71073 Å
Hall symbol: -P 2ac 2ab	Cell parameters from 5654 reflections
*a* = 7.2670 (14) Å	θ = 3.0–27.5°
*b* = 13.741 (3) Å	µ = 0.10 mm^−^^1^
*c* = 19.127 (4) Å	*T* = 93 K
*V* = 1909.9 (6) Å^3^	Prism, yellow
*Z* = 8	0.40 × 0.33 × 0.13 mm

### Data collection

**Table:**

Rigaku SPIDER diffractometer	2019 reflections with *I* > 2σ(*I*)
Radiation source: Rotating anode	*R*_int_ = 0.030
graphite	θ_max_ = 27.5°, θ_min_ = 3.2°
ω scans	*h* = −9→9
14290 measured reflections	*k* = −17→17
2179 independent reflections	*l* = −24→24

### Refinement

**Table:**

Refinement on *F*^2^	Primary atom site location: structure-invariant direct methods
Least-squares matrix: full	Secondary atom site location: difference Fourier map
*R*[*F*^2^ > 2σ(*F*^2^)] = 0.048	Hydrogen site location: inferred from neighbouring sites
*wR*(*F*^2^) = 0.099	H-atom parameters constrained
*S* = 1.00	*w* = 1/[σ^2^(*F*_o_^2^) + (0.0285*P*)^2^ + 1.6*P*] where *P* = (*F*_o_^2^ + 2*F*_c_^2^)/3
2179 reflections	(Δ/σ)_max_ = 0.001
136 parameters	Δρ_max_ = 0.30 e Å^−^^3^
0 restraints	Δρ_min_ = −0.20 e Å^−^^3^

### Special details

**Table:**

Geometry. All e.s.d.'s (except the e.s.d. in the dihedral angle between two l.s. planes) are estimated using the full covariance matrix. The cell e.s.d.'s are taken into account individually in the estimation of e.s.d.'s in distances, angles and torsion angles; correlations between e.s.d.'s in cell parameters are only used when they are defined by crystal symmetry. An approximate (isotropic) treatment of cell e.s.d.'s is used for estimating e.s.d.'s involving l.s. planes.
Refinement. Refinement of *F*^2^ against ALL reflections. The weighted *R*-factor *wR* and goodness of fit *S* are based on *F*^2^, conventional *R*-factors *R* are based on *F*, with *F* set to zero for negative *F*^2^. The threshold expression of *F*^2^ > σ(*F*^2^) is used only for calculating *R*-factors(gt) *etc*. and is not relevant to the choice of reflections for refinement. *R*-factors based on *F*^2^ are statistically about twice as large as those based on *F*, and *R*- factors based on ALL data will be even larger.

### Fractional atomic coordinates and isotropic or equivalent isotropic displacement parameters (Å^2^)

**Table:**

	*x*	*y*	*z*	*U*_iso_*/*U*_eq_	
O1	0.4804 (2)	0.25001 (8)	0.56207 (6)	0.0438 (4)	
O2	0.4755 (2)	0.35241 (8)	0.47651 (6)	0.0414 (3)	
N1	0.50070 (19)	0.33185 (9)	0.53854 (6)	0.0270 (3)	
C1	0.61925 (19)	0.47388 (10)	0.70231 (7)	0.0193 (3)	
C2	0.67974 (19)	0.56401 (10)	0.67959 (8)	0.0216 (3)	
H2	0.6907	0.5759	0.6308	0.026*	
C3	0.7251 (2)	0.63794 (10)	0.72673 (8)	0.0233 (3)	
H3	0.7665	0.6991	0.7097	0.028*	
C4	0.7103 (2)	0.62294 (10)	0.79730 (8)	0.0237 (3)	
H4	0.7403	0.6740	0.8288	0.028*	
C5	0.6346 (2)	0.51571 (11)	0.89674 (8)	0.0250 (3)	
H5	0.6628	0.5669	0.9283	0.030*	
C6	0.5794 (2)	0.42761 (11)	0.92232 (8)	0.0260 (3)	
H6	0.5693	0.4178	0.9713	0.031*	
C7	0.5373 (2)	0.35112 (11)	0.87574 (8)	0.0246 (3)	
H7	0.5001	0.2897	0.8936	0.030*	
C8	0.5497 (2)	0.36480 (10)	0.80489 (7)	0.0216 (3)	
H8	0.5198	0.3126	0.7744	0.026*	
C9	0.60624 (18)	0.45543 (9)	0.77616 (7)	0.0189 (3)	
C10	0.65057 (19)	0.53203 (10)	0.82360 (8)	0.0204 (3)	
C11	0.5682 (2)	0.39854 (10)	0.65200 (7)	0.0219 (3)	
H11	0.5419	0.3356	0.6700	0.026*	
C12	0.5554 (2)	0.41047 (11)	0.58392 (8)	0.0278 (3)	
H12	0.5829	0.4723	0.5642	0.033*	

### Atomic displacement parameters (Å^2^)

**Table:**

	*U*^11^	*U*^22^	*U*^33^	*U*^12^	*U*^13^	*U*^23^
O1	0.0825 (10)	0.0188 (5)	0.0300 (6)	−0.0081 (6)	0.0015 (6)	−0.0001 (5)
O2	0.0744 (10)	0.0281 (6)	0.0216 (6)	−0.0004 (6)	−0.0060 (6)	−0.0006 (5)
N1	0.0370 (7)	0.0208 (6)	0.0231 (6)	0.0009 (6)	0.0016 (5)	−0.0004 (5)
C1	0.0165 (6)	0.0179 (6)	0.0236 (7)	0.0030 (5)	−0.0002 (5)	0.0002 (5)
C2	0.0190 (7)	0.0218 (7)	0.0241 (7)	0.0022 (5)	−0.0003 (5)	0.0033 (6)
C3	0.0191 (7)	0.0169 (6)	0.0339 (8)	−0.0018 (5)	−0.0003 (6)	0.0030 (6)
C4	0.0209 (7)	0.0195 (6)	0.0306 (8)	0.0003 (5)	−0.0013 (6)	−0.0041 (6)
C5	0.0242 (7)	0.0255 (7)	0.0252 (7)	0.0034 (6)	−0.0003 (6)	−0.0054 (6)
C6	0.0281 (8)	0.0285 (7)	0.0214 (7)	0.0056 (6)	0.0031 (6)	0.0010 (6)
C7	0.0259 (7)	0.0209 (7)	0.0270 (7)	0.0040 (6)	0.0052 (6)	0.0048 (6)
C8	0.0223 (7)	0.0174 (6)	0.0250 (7)	0.0030 (5)	0.0011 (6)	−0.0002 (5)
C9	0.0147 (6)	0.0175 (6)	0.0245 (7)	0.0038 (5)	0.0009 (5)	−0.0001 (5)
C10	0.0157 (6)	0.0200 (7)	0.0255 (7)	0.0030 (5)	−0.0002 (5)	−0.0014 (5)
C11	0.0242 (7)	0.0167 (6)	0.0249 (7)	0.0021 (5)	0.0003 (6)	0.0016 (5)
C12	0.0389 (9)	0.0185 (7)	0.0260 (7)	−0.0042 (6)	−0.0012 (7)	−0.0003 (6)

### Geometric parameters (Å, °)

**Table:**

O1—N1	1.2201 (17)	C5—C10	1.422 (2)
O2—N1	1.2335 (16)	C5—H5	0.95
N1—C12	1.4417 (19)	C6—C7	1.411 (2)
C1—C2	1.3841 (19)	C6—H6	0.95
C1—C9	1.4381 (19)	C7—C8	1.371 (2)
C1—C11	1.4613 (19)	C7—H7	0.95
C2—C3	1.398 (2)	C8—C9	1.4220 (19)
C2—H2	0.95	C8—H8	0.95
C3—C4	1.370 (2)	C9—C10	1.4265 (19)
C3—H3	0.95	C11—C12	1.316 (2)
C4—C10	1.415 (2)	C11—H11	0.95
C4—H4	0.95	C12—H12	0.95
C5—C6	1.366 (2)		
			
O1—N1—O2	123.23 (13)	C7—C6—H6	120.1
O1—N1—C12	120.13 (13)	C8—C7—C6	120.51 (14)
O2—N1—C12	116.64 (12)	C8—C7—H7	119.7
C2—C1—C9	119.15 (13)	C6—C7—H7	119.7
C2—C1—C11	120.51 (13)	C7—C8—C9	121.40 (13)
C9—C1—C11	120.34 (12)	C7—C8—H8	119.3
C1—C2—C3	121.50 (13)	C9—C8—H8	119.3
C1—C2—H2	119.3	C8—C9—C10	117.75 (13)
C3—C2—H2	119.3	C8—C9—C1	123.58 (13)
C4—C3—C2	120.52 (13)	C10—C9—C1	118.67 (13)
C4—C3—H3	119.7	C4—C10—C5	120.94 (13)
C2—C3—H3	119.7	C4—C10—C9	119.65 (13)
C3—C4—C10	120.48 (13)	C5—C10—C9	119.42 (13)
C3—C4—H4	119.8	C12—C11—C1	125.55 (13)
C10—C4—H4	119.8	C12—C11—H11	117.2
C6—C5—C10	121.07 (14)	C1—C11—H11	117.2
C6—C5—H5	119.5	C11—C12—N1	121.46 (14)
C10—C5—H5	119.5	C11—C12—H12	119.3
C5—C6—C7	119.84 (14)	N1—C12—H12	119.3
C5—C6—H6	120.1		
			
C9—C1—C2—C3	−1.2 (2)	C3—C4—C10—C5	−179.96 (14)
C11—C1—C2—C3	178.42 (13)	C3—C4—C10—C9	0.2 (2)
C1—C2—C3—C4	−0.1 (2)	C6—C5—C10—C4	−179.12 (14)
C2—C3—C4—C10	0.6 (2)	C6—C5—C10—C9	0.7 (2)
C10—C5—C6—C7	0.0 (2)	C8—C9—C10—C4	179.02 (12)
C5—C6—C7—C8	−0.7 (2)	C1—C9—C10—C4	−1.6 (2)
C6—C7—C8—C9	0.5 (2)	C8—C9—C10—C5	−0.79 (19)
C7—C8—C9—C10	0.2 (2)	C1—C9—C10—C5	178.64 (13)
C7—C8—C9—C1	−179.20 (14)	C2—C1—C11—C12	−7.4 (2)
C2—C1—C9—C8	−178.57 (13)	C9—C1—C11—C12	172.20 (15)
C11—C1—C9—C8	1.8 (2)	C1—C11—C12—N1	−178.90 (14)
C2—C1—C9—C10	2.03 (19)	O1—N1—C12—C11	−6.4 (2)
C11—C1—C9—C10	−177.61 (12)	O2—N1—C12—C11	173.19 (16)

### Hydrogen-bond geometry (Å, °)

**Table:**

*D*—H···*A*	*D*—H	H···*A*	*D*···*A*	*D*—H···*A*
C2—H2···O2^i^	0.95	2.58	3.3919 (19)	144
C7—H7···O2^ii^	0.95	2.52	3.4261 (19)	159
C12—H12···O2^i^	0.95	2.57	3.464 (2)	158

Symmetry codes: (i) −*x*+1, −*y*+1, −*z*+1; (ii) *x*, −*y*+1/2, *z*+1/2.
